# Fine Tuning of an Advanced Planner for Cognitive Training of Older Adults

**DOI:** 10.3390/ejihpe15010004

**Published:** 2025-01-07

**Authors:** Mauro Gaspari, Giovanna Mioni, Dario Signorello, Franca Stablum, Sara Zuppiroli

**Affiliations:** 1 Department of Computer Science and Engineering, University of Bologna, 40126 Bologna, Italy; sara.zuppiroli@unibo.it; 2 Department of General Psychology, University of Padova, 35131 Padova, Italy; giovanna.mioni@unipd.it (G.M.); dario95sign@gmail.com (D.S.); franca.stablum@unipd.it (F.S.); 3 Human Inspired Technology Research Centre, University of Padova, 35121 Padova, Italy; 4 CNR—Institute of Cognitive Sciences and Technologies, 40121 Bologna, Italy

**Keywords:** cognitive training, planning, executive functions, user studies, heuristic search planner

## Abstract

Developing effective cognitive training tools for older adults, specifically addressing executive functions such as planning, is a challenging task. It is of paramount importance to ensure the implementation of engaging activities that must be tailored to the specific needs and expectations of older adults. Furthermore, it is essential to provide the appropriate level of complexity for the planning task. A human-centred approach was used to address the issues identified in the design of the tool. Two pilot studies were conducted with older adults to fine-tune the training task and optimize its suitability for them. This also led to an enhancement of the underlying planning engine, transitioning from a simple fast-forward planner (PDDL4J) to an advanced heuristic search planner (ENHSP). The results show that user studies enabled the development of a cognitive training system that gradually increased the proposed difficulty levels of the planning task while maintaining usability and satisfaction among older adults. This highlights the importance of conducting user studies when implementing cognitive training tools for older adults.

## 1. Introduction

The world is currently undergoing a profound demographic shift, moving from a population structure in which the majority of individuals were relatively young to one in which a significant proportion of people are over the age of 65. According to data from World Population Prospects: the 2022 Revision (United Nations, 2022), by 2050, one in six people in the world will be over the age of 65 (16%), with this figure rising to one in four for those living in Europe and Northern America. In 2018, for the first time in history, people aged 65 or above outnumbered children under five years of age worldwide. The number of people aged 80 years or over is projected to triple, from 143 million in 2019 to 426 million in 2050 (United Nations, 2024). This change presents both a challenge and an opportunity for the design of intelligent technology for aging (Shishehgar et al., 2018). Cognitive health is an important factor in determining the functional ability of older adults (Beaton et al., 2015; Bezdicek et al., 2021; Gross et al., 2011), and is of paramount importance for maintaining autonomy.

The development of cognitive training programs is becoming a priority for reducing the impact of aging on quality of life. One of the key aspects of successful aging is the ability to solve everyday problems encountered in daily life. Any task that requires planning, organization, memorization, time management, and flexible thinking is particularly challenging for older adults. Retirement and withdrawal from productive activities often lead older people to limit activities and refrain from using problem-solving skills as previously performed. Consequently, people may encounter greater difficulty in finding a successful solution to a problem as they age. Previous studies have indicated that cognitive training, even when initiated later, can have positive benefits, with reduced rates of cognitive decline and a lower incidence of dementia (Beydoun et al., 2014; Geda et al., 2012).

Based on this evidence, brain games, initially available in a paper-and-pen format, have been designed and implemented on computers to train problem-solving abilities (Burgess, 1997; Goghari & Lawlor-Savage, 2017; Manera et al., 2015; Rose et al., 2015; Zinke et al., 2014). To be effective, training tasks should have high ecological validity (training participants to perform typical activities of daily life), be easily usable, and be sufficiently engaging (Diamond & Ling, 2016; Leung et al., 2022; Moreau & Conway, 2014). This can minimize the number of people who abandon the training, thereby increasing the number of individuals who can benefit from the training. For example, an engaging scenario can be designed to simulate a visit to a historic city with several constraints and goals to achieve within a limited timeframe. However, this kind of ecological task requires more complex solutions, such as tailoring exercises to the needs of older adults and defining progressively increasing difficulty levels. As clearly stated by Diamond and Ling (2016) executive functions need to be continually challenged to see improvements (see also Ericsson, 2017; Lövdén et al., 2010; Moreau & Conway, 2014; Simon et al., 2018). Currently, most proposed training solutions use ad hoc approaches in which the same scenario is repeated at a fixed difficulty level.

The primary theoretical issue with ad hoc solutions is the practice effect that arises when the same training task is performed on multiple occasions. Older adults may rely on their previous experience to solve the proposed problems, which can potentially hinder the training of real-life problem-solving abilities. A crucial feature is the novelty of the task (Burgess, 1997; Randolph & Chaytor, 2022). The formulation of a plan is essential when confronted with novel tasks. This plan should comprise the appropriate sequence of behaviors required to achieve the desired outcome. Plans should be compared in terms of their relative probability of success and their relative efficiency in achieving the chosen goal. Plans should then be initiated and subsequently implemented, with amendments made as required until success is achieved or until the imminent failure of the plan is recognized. The design of planning and problem-solving training is frequently constrained by the complexity of the generation of novel goals and tasks. In many cases, only a limited number of scenarios with restricted variability are employed.

The use of automatic planning may prove to be an effective approach for the generation of novel scenarios. For example, automatic planning has been shown to be beneficial in crafting realistic and engaging scenarios with human-centered features (Baschieri et al., 2018; Gaspari et al., 2023). This approach allows the generation and assessment of a diverse range of training tasks that can be tailored to the specific capabilities and constraints of older adults (Ziegler et al., 2022), thereby providing opportunities for them to develop and hone their problem-solving abilities. AI technologies enable the development of a multitude of open problem-solving tasks that closely resemble real-life scenarios and are designed to be solvable. In addition, the use of automatic planning can be helpful in the production of training tasks with progressively higher levels of difficulty. In fact, the time a planner requires to find a solution, as well as the length of this solution, can be used to estimate the actual difficulty of the proposed exercises.

In summary, from a technological perspective, the design of a cognitive training system based on planning that supports the aforementioned features poses several challenges:

- The design of engaging problems for older adults;
- An accurate determination of the appropriate difficulty levels of the exercises;
- The design of a mechanism to adapt the difficulty of exercises to subjects throughout the training.

To address these challenges, it is essential that the design and implementation of cognitive training tasks involve older adults. This should be conducted in a way that builds a solution specifically conceived for them, following a participatory design approach that provides further theoretical underpinning.

The objective of this research is to address the above challenges through a complex training task developed as part of the SWIFT (Shared, Web-based, Intelligent Flexible Thinking Training) project. The project aims to develop a framework to support problem-solving training for older adults. The SWIFT framework consists of a platform that provides a set of training tasks, a user interface for older adults, and one for administrators, enabling them to configure and monitor training sessions.

The proposed task requires users to plan a two-day vacation in a European city (Rome). In this scenario, it is of paramount importance to adapt the difficulty of the exercises to the subjects undergoing training (Ziegler et al., 2022), as well as to determine the appropriate difficulty level of the exercises in question. These goals should be achieved while maintaining the requisite standards of usability and ecological appearance.

The contribution of this paper is to emphasize the pivotal role of user studies in fine-tuning the difficulty levels of a complex planning task to adapt to older adults. We illustrate how the aforementioned challenges can be addressed through refinement, achieved via two primary pilot studies employing both quantitative and qualitative research methods. Pilot Study A was conducted to evaluate the usability and satisfaction of the tool and to identify performance differences between young and older adults. Pilot Study B was designed to test the difficulty stages, usability, and effectiveness of the tool specifically for older adults.

Following a brief review of the relevant literature, this paper presents the methodology used in the development process, the proposed task, the application of automated planning, and the two pilot studies, concluding with a final discussion.

## 2. Literature Review

Pollack (2005) identifies three classes of systems that employ artificial intelligence (AI) techniques to support older adults. The first class comprises systems that monitor an individual and generate alerts and status updates. The second class of systems is designed to assist older adults in compensating for cognitive impairments. Such systems can facilitate the management of daily schedules, the completion of multi-step tasks, the recognition of faces, and the localization of objects. The third class of systems employs AI to provide a continuous assessment of the cognitive state of older adults. The systems reported by Pollack represent only a subset of possible applications; in fact, AI can also be used to predict or support older adults who experience cognitive decline (Graham et al., 2020). Another noteworthy application is the use of AI techniques to enable older adults to exercise their abilities and to enhance them, for example, to improve human decision making (Callaway et al., 2022). Exercises are serious games that may involve physical activities, cognitive activities, or both (Eun et al., 2023).

A number of reviews and meta-analyses have investigated the impact of executive function training on older adults (see, for example, Diamond & Ling, 2016, 2020; Kelly et al., 2014; Stojan & Voelcker-Rehage, 2019). With consideration of the specific focus of our study, we restricted the scope of our analysis to computer programs designed to enhance planning skills. Our concise review excludes virtual reality studies, which represents a discrete area of inquiry. Moreover, although some studies, such as the recent work presented in Eun et al. (2023), utilize artificial intelligence techniques, they typically encompass physical activities and do not prioritize planning as a cognitive factor.

With regard to planning, both experimental and commercial systems provide ecological tasks to train planning abilities. The Plan-A-Day approach (Holt et al., 2011), the Game of Gifts Purchase exercise presented in (López-Martínez et al., 2011), or the shopping exercise implemented in the Rehacom cognitive training system (Benham et al., 2022) can be cited as examples. However, the majority of the proposed tasks adopt ad hoc solutions. To illustrate, the implementation of Plan-A-Day, as presented in (Holt et al., 2011), offers only eight fixed problems with increasing difficulty levels. Furthermore, performance is evaluated on the solution time rather than the correctness of the plan. In this context, the use of AI technology, such as automated planning, for serious games and training tasks has commenced over the past decade, as evidenced in the literature (Baschieri et al., 2018; Do & Tran, 2013; Gaspari et al., 2023; Menif et al., 2013). However, none of the proposed exercises reaches the complexity of the Weekend in Rome task, which is inherently more challenging than the aforementioned examples. It encompasses multiple days and combines various activities.

Other studies specifically focused on computerized training of planning skills (Goghari & Lawlor-Savage, 2017; Manera et al., 2015; Wang et al., 2011; Zinke et al., 2014) have been reported in the literature, but there is no evidence of the use of artificial intelligence techniques in the implementation of training tasks.

In (Zinke et al., 2014), the Tower of London Task is presented. It requires moving five differently colored balls on a board with three pegs from a start position to an end position. The task is designed to increase in difficulty as the number of required moves to solve the problem is increased from three to eleven.

In the Goghari and Lawlor-Savage (2017) study, the logic and planning training games were provided by BrainGymmer “https://www.braingymmer.com/en/ (accessed on 19 November 2024)”. The adaptation criteria were based on error thresholds. Three distinct games were selected. In the Square Logic game, a grid of numbered squares was presented. The objective was to arrange the squares in accordance with the rule that squares can only be stacked onto squares that are one point higher or lower in value. In the Out of Order game, a series of squares was presented, each with different shapes, patterns within the shape, color, and number of shapes. The objective of this game was to rearrange the squares so that each square matched at least one characteristic of the square adjacent to it. The Patterned Logic involved a pattern with missing pieces, which participants had to choose and complete.

In the Manera et al. (2015) study, a serious game was presented for the assessment and rehabilitation of older people with mild cognitive impairment (MCI), Alzheimer’s disease (AD), and related disorders. The Kitchen and Cooking game was born from the tight collaboration between clinicians and game designers and was developed to assess and stimulate planning abilities and praxis. The game is based on a cooking plot, where participants can play different scenarios/recipes: pizza, yoghurt cake, chicken breast in cream sauce, and salmon wrap. In each scenario, participants need to select the correct ingredients from the fridge and cupboards, plan which actions need to be performed, and in which order, and perform specific gestures to accomplish each action. Depending on the scenario, the number of objects to be recognized ranges from 5 to 7, the number of executive function activities ranges from 5 to 8, and the number of praxis ranges from 7 to 13.

Wang et al. (2011) gave older adults training in a computerized task related to real life, cooking a meal. Participants constantly switched, updated, and planned to control the cooking of several foods and concurrently performed a secondary task of setting the table. The training task was divided into five speed levels by varying the speed of the timer, and the complexity of the task was also varied on the basis of the numbers of dishes (from 2 to 6).

In conclusion, the previously implemented tasks were limited in scope, encompassing only a restricted set of predefined issues and solutions. The introduction of artificial intelligence techniques, such as automatic planning, has the potential to facilitate the generation of numerous novel scenarios and permit the implementation of a diverse array of alternative optimal resolutions. This reflects the characteristics typically observed in real-life planning activities, whereby a multitude of potential scenarios and solutions must be considered.

## 3. Method of the Development Process

The design of ecological training tasks for executive functions requires a coordinated multidisciplinary research effort. On the one hand, sophisticated technical solutions are required, such as the use of automated planning techniques for the generation of exercises or the adaptation of exercises to the requisite level of difficulty. On the other hand, the supervision of cognitive psychologists is of paramount importance. Testing with subjects is essential for tuning tasks before delivery. It is also essential to consider issues such as personalization and adaptability when working with older adults (Lu et al., 2017). Indeed, the reduced plasticity in aging requires a higher level of customization and adaptability.

**Figure 1:**
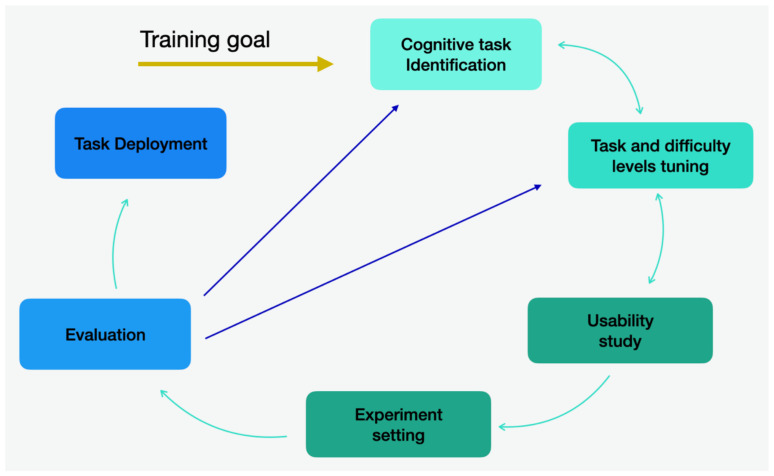
Graphical representation of the cyclic development process.

The cyclic development process is depicted in Figure 1. The development of tasks is divided into six macro-phases based on a cyclic structure. The sequence of the phases is not fixed; movement between them is possible in both directions. The outcome of each phase determines which phase to be performed next. A working version of the software is produced during the first step, so experimentation can begin early in the software life cycle. Each subsequent release of the task incorporates new functions or rectifies any deficiencies present in the previous release.

In the course of our development process, we employed a participatory design.

The identification of the task began with an initial prototype of a cognitive training task, named Weekend in Rome, referred to as Version 0.0 (V0.0), which required users to plan a two-day vacation in Rome (Gaspari & Donnici, 2019). Subsequently, this prototype was enhanced (V1.0) through the participation of older adults in focus groups (Cipolletta et al., 2024), with the aim of addressing fundamental requirements.

The focus group study confirmed that planning a two-day vacation would be an engaging task for older adults, and allowed us to gather requirements from them, such as considering the budget, train and hotel booking, the type and number of places to visit, eating, and the importance of making daily plans. Subsequently, pilot studies A and B were conducted. Pilot study A assessed user satisfaction, which allowed the prototype to be refined (V2.0). Pilot study B was then carried out to assess the system’s effectiveness and gather preliminary results.

### 3.1. The Weekend in Rome Task

In the Weekend in Rome task, users have to organize virtual train and hotel reservations and complete various activities, such as visiting specific locations and attending particular events. To execute these tasks, users have to navigate a map where the goals are those typically encountered in the real-life planning of trips (e.g., making reservations, checking bus schedules, and noting opening hours of specific locations). This scenario is encoded as a planning problem using PDDL (Planning Domain Definition Language) (Haslum et al., 2019). This approach enables the generation of numerous instances of the problem, each with different goals and constraints. This is possible because the planner can be used to assess the feasibility of each instance.

**Figure 2:**
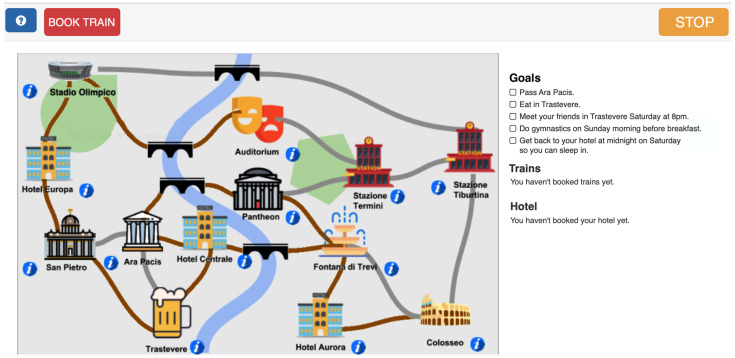
Example of the medium stage. The user interface for the Weekend in Rome task is designed to be simple and not confusing for older users. It presents a map of the city, goals, and some buttons that appear or disappear dynamically to facilitate its use. The user can navigate the map using the mouse by clicking on POIs adjacent to the one where they are.

The system proposes three main stages of difficulty, designated as easy, medium, and difficult. Each stage comprises at least three distinct instances of the problem, each of which must be solved twice in order to advance to the subsequent level. The easy stage is characterized by a map in which each point can only be reached on foot; there are eight points of interest (henceforth referred to as POIs) placed on the map, and the user is required to solve from a minimum of three goals to a maximum of five. In the medium stage, a map is presented where some connections are possible only with the use of buses. Buses operate on a scheduled basis, with specific times of operation indicated on the map. Additionally, the map includes a second railway station, from which users can embark or disembark. An illustrative example of this stage is presented in Figure 2. In the medium stage, users are required to achieve a number of goals, ranging from a minimum of six to a maximum of eight. In the difficult stage, a new POI is added to the map and users are asked to achieve a minimum of seven and a maximum of ten goals. For each stage, three instances of the task are provided with increasing difficulty levels. In order to complete their training, users must finish all difficulty stages, consisting of nine tasks, planning their journey by achieving at least 80% of the goals in each task.

Three types of goals can be achieved: a simple passage from a POI (e.g., visited Pantheon); a visit at a POI, which must take place within the opening hours of the attraction (e.g., done-activity Colosseum); a visit at a POI at a given time, for doing a specific activity (e.g., done-activity-timed Olympic Stadium at 18). Although the developed exercise is specific to Rome, the structure can be implemented for any European city.

### 3.2. Exploiting Automated Planning

Versions 0.0 and 1.0 of the Weekend in Rome prototype were based on an automatic planner, PDDL4J (Planning Domain Description Library for Java) (Pellier & Fiorino, 2018). The planning domain is described using PDDL 1.2, which also allows the specification of several problems to be solved dynamically.

The planning domain encodes a set of PDDL rules, which encompasses all possible actions and interactions with the user. These include travel (e.g., walking, bus, and train), activities to be carried out at a POI (e.g., visiting, visiting at a certain time), sleeping and having breakfast in a booked hotel, and exercising. A planning problem, in accordance with the specified difficulty stage, incorporates the specific activities, bus and train timetables, connections between the various points on the map, and the goals to be achieved.

**Figure 3:**
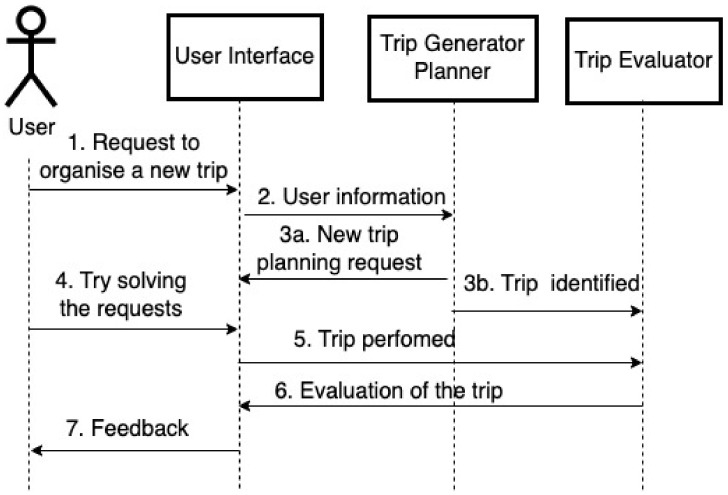
The interaction between the user and the planner.

The planner is used in different phases of the training process, namely, for the generation of new solvable exercises and for the evaluation of solutions. The interaction between the user and the planner is depicted in Figure 3. The Trip Generator is activated when the user is required to undertake a new instance of the task (1). It takes as input the user profile and the level of difficulty of the new task (2), and generates a new problem instance by extracting the goals to be achieved by the user from a set of possible goals at random (3a). Successively, the Trip Generator calls the planner to find a plan that solves the new instance of the problem (3b). If the planner fails, steps (3a) and (3b) are repeated, and other goals are selected until a solvable scenario is created (3b). Once a solvable scenario is generated, the user can start to execute the task (4). At the end of the exercise, upon the user’s completion of their visits by taking the return train (5), another component is initiated, the Trip Evaluator (6). The Trip Evaluator quantifies the number of goals achieved, assigning a percentage rating to the user ranging from 0 to 100. A difficulty level is deemed to have been completed successfully when the obtained percentage is at least 80%. Consequently, it is possible to pass the instance of the problem even if the plan has not been fully executed. Additionally, the Trip Evaluator provides feedback to the user on the plan implemented (7). For example, “Congratulations! You completed this exercise without any errors” when a difficult exercise is passed, or “This exercise was much more difficult than the previous one. Try to keep track of bus schedules” in case of failure.

## 4. Pilot Study A: Testing Usability and Satisfaction

Pilot study A was conducted with two groups of participants: a group of healthy young adults and a group of older adults.

The pilot study had the following aims:

1. To provide a preliminary validation of the usability of the training task on healthy older adults;
2. To identify the specific requests and needs of older adults when performing the task;
3. To identify processing characteristics specific to older adults by examining differences in performance between older and younger adults;
4. To test the difficulty stages proposed by the system;
5. To collect all the relevant suggestions proposed by the participants.

### 4.1. Methods

A total of 22 participants were recruited for the study, comprising 11 young adults (aged 18 to 26 years) and 11 older adults (aged 62 to 83 years). The young adult group, which consisted of three males and eight females, had an average age of 23.64 (SD = 1.12) and an average of 17.27 years of education (SD = 1.01). The older adult group, comprising six males and five females, had an average age of 68.73 (SD = 7.55) and an average of 11.45 years of education (SD = 1.96).

All participants completed a series of 40-minute training sessions until they had experienced all difficulty levels. Prior to the commencement of the study, all participants signed the Research Informed Consent Form and received written instructions for accessing and utilizing the training tool. Young adults completed the online sessions independently, while older adults were supervised until they demonstrated satisfactory compliance with the tool. All participants were instructed to contact the experimenter if they required further information or clarification. The sessions were monitored using remote control facilities provided by the system. At the conclusion of the sessions, all participants completed a usability questionnaire.

### 4.2. Results

The results of the usability questionnaire indicated that all older participants were able to easily access the online system. Furthermore, the majority of them (9/11) reported no difficulty in understanding and performing the task. The responses were based on a Likert scale (1 = not at all; 4 = a lot). Most older participants indicated that good planning abilities (M = 3.64; SD = 0.50) and computer experience (M = 3; SD = 0.63) were crucial to complete the task. Regarding the gradual increase in difficulty, the responses indicated limited satisfaction (M = 2.45; SD = 0.93). Furthermore, the item related to the ecological quality of the task indicated a need for improvement (M = 2.73; SD = 0.90). Older adults identified high involvement, the possibility for improving their problem-solving and planning abilities, the engaging and challenging task format, and the new technological approach as the main strengths of the task. Older participants offered several suggestions for improvement. These included making the task goals visible on the map at all times, streamlining the train booking process, adding new places to visit, changing the color of the streets in the map to enhance visibility, and adding new actions related to a real journey (i.e., the introduction of a budget for the trip to cover expenses of hotels, trains, and buses). All participants successfully completed the task.

A *t*-test was conducted on the critical dependent variables, with group as the between-subject factor (young vs. older). The following performance variables were evaluated: number of sessions, number of not-achieved goals, execution time (minutes), number of clicks on reservations, and number of clicks on goals (see Table 1). The established threshold for significance was *p* < 0.05. A significant difference was found between the two groups in the number of sessions, execution time, and the number of clicks on reservations. The older adults showed a higher number of sessions, a longer execution time, and a higher number of clicks on the reservation, indicating that they checked the train and hotel reservations more often.

**Figure 4:**
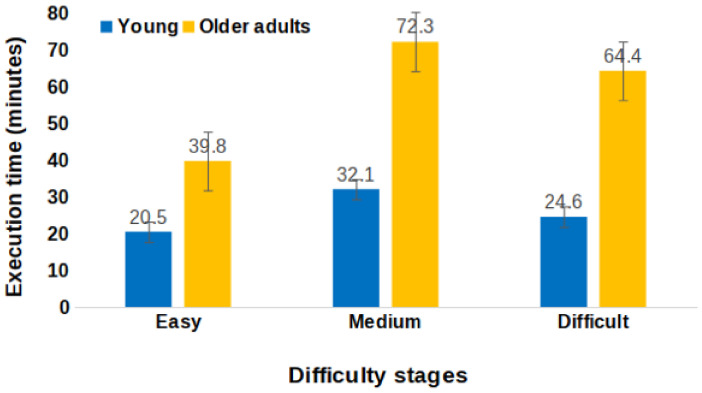
Means and standard deviations of the execution time (in minutes) in the easy, medium, and difficult stages for young and older adults.

To test the difficulty stage proposed by the Trip Generator, a repeated-measures ANOVA was carried out on execution time. The between-subject factor was group (young and older adults), while the within-subject factor was difficulty stage (easy, medium, and difficult). The simple effects of group (F (1, 20) = 20, *p* < 0.001) and difficulty stage (F (2, 40) = 17.43, *p* < 0.001) were significant. It is noteworthy that the interaction between group and difficulty stage was significant (F (2, 40) = 5, *p* = 0.012) (see Figure 4). To better understand the source of this interaction, planned comparisons were performed. Older adults were slower than younger adults for all difficulty stages. Furthermore, older adults were slower in the medium and difficult stages relative to the easy stage. Interestingly, no significant differences emerged between the medium and difficult stages for both young and older participants.

### 4.3. Discussion: Enhancement of the Training Task

The results of pilot study A allowed the identification of several areas for improvement in the training task, particularly in relation to the use of automated planning.

With regard to usability, pilot study A confirmed that the user interface was well designed and was not confusing for older participants. Nevertheless, in response to the suggestions of the participants, several modifications were implemented. These included improvements to map visibility, the display of task goals, and train reservation procedures. In addition to conventional trains, the latter now include high-speed trains. To this end, a new panel was added in the interface to the right of the map presented in Figure 2, eliminating the need for repeated clicks on the reservations and goals buttons. Another suggestion was to make the task more similar to a real journey. To this end, new locations to visit were incorporated, short videos were created for specific POIs to present general information and their history, and new actions to be completed were added.

However, several comments were not related to simple updates of the user interface, but, rather, had a strong implication on the system architecture, for example, the introduction of a limited budget for the trip to cover expenses of hotels, trains, and buses. This new feature affected both the planner and the user interface, the latter with the introduction of a spreadsheet for expenses and the simulation of credit card payments.

Taking into account the planner, a key objective was to test the progression of difficulty stages, which was based on nine increasing difficulty levels (three for each stage).

In Version 1.0 of Weekend in Rome, the progression of difficulty levels was based on increasing the number of goals tied to specific times and introducing, from easy to intermediate levels, the presence of buses that allowed travel between points on the map only at certain times. These constraints led to a reduction in the number of possible plans for solving the problem and an increase in the number of steps required to solve the plan. The planning problem was encoded in PDDL 1.2, where time and movements were managed by predicates, and the actions the user could perform on the map were described by actions in the domain.

Although the introduction of buses from easy to medium stages allowed an adequate increase in difficulty, we did not observe the same effectiveness in simply introducing a greater number of goals during pilot study A. Upon analysis of the results, it became evident that an increase in the difficulty stage did not always correspond to an increase in the difficulty of the user. More precisely, it was observed that when moving from the medium to the difficult stage, merely increasing the difficulty of the activities with more stringent time constraints and/or adding new goals did not necessarily result in an increase in the real and perceived difficulty: participants completed the training for the medium and the difficult stages in the same execution time. It became evident that the progression of difficulty stages in the final part of the exercise was not as steep as it could have been. In essence, the time required to solve these tasks and the number of attempts to pass them decreased on average, whereas an increase in both was expected and more appropriate for cognitive training. It was determined that this effect was caused by the rule used for passing to the next difficulty level, which applies when an 80% performance is obtained. The issue was that the minimum number of goals required to achieve the threshold was not changing in accordance with the progression of difficulty stages.

To address this issue, the rule for advancing to the next level was updated, requiring participants to achieve all the proposed goals and execute the plan without errors. Moreover, additional goals and constraints were introduced to further reduce the number of possible plans to reach the correct solution. These included requirements to minimize the cost of the trip or the time spent in the city at the difficult stage. The introduction of the budget variable was intended not only to enhance the ecological value of the game, but also to increase the difficulty of the exercises. At the difficult stage, three minimization objectives were identified: one on time, one on costs, and one encompassing both time and travel costs.

**Figure 5:**
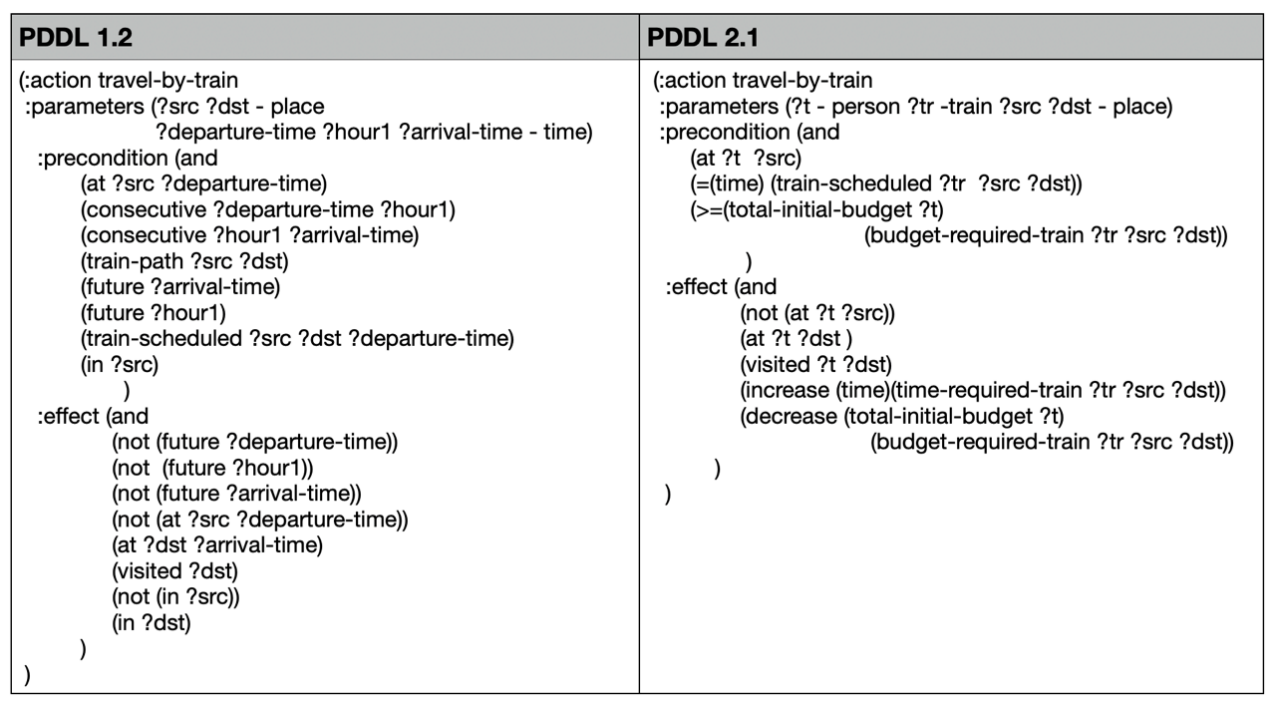
The implementation of the “travel-by-train” action, comparing PDDL 1.2 with PDDL 2.1 (variables start with a question mark).

Since these features were not supported by the planner used in versions 0.0 and 0.1 (PDDL4J), which only supports PDDL 1.2, it became necessary to rewrite the domain using PDDL 2.1, introducing functions for the representation of time and budget. Consequently, an upgrade was implemented with the Expressive Numeric Heuristic Search Planner (ENHSP) (Scala et al., 2016), which supports fluents and plan metrics as required by PDDL 2.1. Figure 5 provides an illustration of the transition from PDDL 1.2 to 2.1. It presents the encoding of the action “travel-by-train”, which implements the train trip to Rome, utilizing budget and time as fluents. The definition of the planning domain was improved by employing a more expressive language. Indeed, several predicates were required to implement the progression of time in PDDL 1.2. These were used to represent that two time instants are consecutive and to state that they are not in the future anymore when the action is executed. In contrast, in PDDL 2.1, increasing the time variable was sufficient.

## 5. Pilot Study B: Testing Difficulty Stages, Usability, and Effectiveness

A new version of the Weekend in Rome task (V2.0), including all the illustrated changes, was delivered. Subsequently, pilot study B was designed to test the progression of the updated difficulty stages, the usability of the improved version, and to gather preliminary effectiveness results. This second pilot study (B) involved only a cohort of healthy older adults.

The objectives of this pilot study were as follows:

1. To assess the actual increase in difficulty compared to the previous version;
2. To validate the usability of the system, including the collection of suggestions and the assessment of participant satisfaction;
3. To assess the improvement in ecological appearance;
4. To test whether there are improvements in the trained cognitive ability, specifically planning and problem-solving skills;
5. To assess the trained cognitive abilities three months after training.

### 5.1. Methods

The study comprised a sample of 22 participants (aged 67–81 years), divided into an experimental group and a control group. The selection criteria for the participants in both groups were as follows: individuals aged 65 and above, with no cognitive and/or psychiatric disorders. The experimental group, which consisted of seven males and four females, had an average age of 72.72 years (SD = 4.90) and an average of 12.36 years of education (SD = 5.20). The control group, consisting of six males and five females, had an average age of 70.18 (SD = 4.35) and an average of 13.65 years of education (SD = 3.75). Before starting the study, all participants signed the Research Informed Consent Form. The experimental group received written instruction on how to access and use the training tool. The training phase was delivered exclusively to the experimental group and comprised eight training sessions (two per week), each lasting 40 min, using the Weekend in Rome task (V2.0). The training sessions were monitored, with 10 out of 11 participants being observed in person and one remotely using the facilities provided by the SWIFT platform.

Participants were assessed at three distinct time points: T1, the test phase, at the beginning of the study, to establish baseline performance; T2, the retest phase, soon after training, five weeks after the test phase; and T3, the follow-up phase, three months after the retest phase, exclusively among the experimental group. The assessments were administered to all participants in the Department of General Psychology (Padova). The following tests were administered: the Behavioural Assessment of the Dysexecutive Syndrome (BADS) (Antonucci et al., 2014; Wilson et al., 1996) and the Everyday Problem Test (EPT) (Borella et al., 2017; Willis & Marsiske, 1993).

The BADS is a battery for the assessment of executive functions, comprising six subtests. The Rule Shift Cards Test assesses the ability to inhibit a previously learned response mode. This test is designed to assess cognitive flexibility. The Action Program Test assesses the ability to develop an action plan to solve a problem. The Key Search Test assesses the ability to plan actions and monitor one’s performance. The Temporal Judgment Test assesses the ability to predict and estimate time. The Zoo Map Test assesses the subject’s ability to plan and minimize errors by self-monitoring. The Modified Six Elements Test assesses the subject’s organizational ability, shifting ability, and behavioral control. Each test is associated with a specific scoring method and is calibrated to establish cut-offs based on the age of the participant and the execution time. The EPT is a test of everyday problem-solving, with a focus on performance accuracy. It presents real-world problems that cover all seven instrumental activities of daily living domains (household management, transportation, meal preparation and nutrition, financial management, health, shopping, and telephone skills). The abbreviated (14-item) and parallel (14-item) versions of the Italian adaptation of the test were employed. One point is awarded for a correct answer, while zero points are given for an incorrect response. Subsequently, the scores are adjusted according to age and educational level cut-offs.

Furthermore, usability and satisfaction questionnaires were administered at the conclusion of each training sessions, as was the case in study A. Additionally, participants were invited to provide suggestions regarding potential modifications to enhance the training task and the SWIFT platform user interface through interviews.

### 5.2. Results

Although the duration of the proposed training was limited to eight sessions, the results of this study yielded several insights. The primary findings pertain to the significant enhancement in the degree of difficulty observed with respect to the initial Weekend in Rome prototype. To this end, we compared the data obtained from the two groups of older adults who underwent training sessions in pilot studies A and B. The results are summarized in Table 2.

Table 2a presents the minimum solution time to solve an exercise at each level of difficulty and the number of older adults who reached a given level. The data in study A indicate that 10 out of 11 participants reached the highest difficulty levels. On the contrary, in study B, only three participants were able to execute the training task at level 7, which is the first level of the difficult stage. This finding demonstrates that modifications made to increase the difficulty level, such as introducing a spending budget, dinner and lunch goals, path minimization, and the requirement to execute the plan without errors, made the exercise more challenging. This is also confirmed by the increase in the time required to solve exercises at a given level. In the revised version of Weekend in Rome (study B), with the exception of the transition between the first and second difficulty levels, where a learning-related effect can be observed, the progression of difficulty levels is monotonic. Consequently, a notable enhancement was achieved compared to study A, where a flattening effect of the minimal time required to solve exercises can be observed.

Furthermore, Table 2b presents the time required to generate new problems at different levels. These data provide another indicator to measure whether the difficulty of the proposed tasks is effectively increased. The Trip Generator always calls the planner to verify that the newly generated exercises are, indeed, solvable. Thus, if solution plans take longer and are more difficult to find, they are presumably more challenging for users.

To test the extent of the enhancement in performance subsequent to the training intervention, separate repeated-measures ANOVAs were carried out on BADS total scores, as well as the BADS subtests scores, and EPT scores only for the experimental group. The within-subject factor was time (test, retest, follow-up). The simple effect of time did not reach the significance level of *p* < 0.05 (see Table 3).

The main results of the assessment are presented in Table 3, which displays the T1, T2, and T3 BADS total scores, as well as the BADS subtests scores, and EPT scores. The table shows that the improvement observed at T2 in the retest phase was nearly lost at T3 in the follow-up phase.

**Figure 6:**
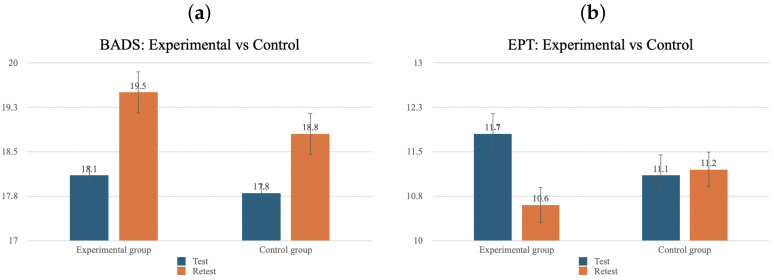
Comparison of the results obtained for the experimental and control groups for the BADS (**a**) and EPT (**b**) total scores at test and re-test. The significant improvement for the experimental group on the BADS total score can be observed on the left.

Furthermore, a comparison of the results of the experimental group with those of the control group was carried out (see Figure 6). Separate repeated-measures ANOVAs were conducted on the BADS and EPT scores. The between-subject factor was group (experimental and control group), while the within-subject factor was time (test, retest). No interaction effects reached the significance level. The BADS total score showed a significant simple effect of time (F (1, 20) = 11.28, *p* = 0.003). Planned comparisons revealed a significant improvement only for the experimental group (*p* = 0.048). The effectiveness data are inconclusive. To enhance the reliability of the training results, it would be prudent to expand the size of the two groups. Additionally, the T1 data indicate a ceiling effect, which implies that the selected tests may have been too straightforward for the participants to demonstrate a change in performance.

### 5.3. Discussion

A noteworthy observation from the results of pilot study B is that data on the training exercises at the last two levels could not be obtained. This may be mainly due to the limited duration of the training, which was restricted to eight sessions only. However, the higher difficulty level of the proposed exercises in the revised version of Weekend in Rome is also corroborated by the reporting of the time required to generate new problems at different levels, as shown in Table 2b. Indeed, the generation of exercises also involves several calls to the planner, which verifies that the newly generated exercises are effectively solvable. Therefore, if the solution plans are longer and more difficult to find, it can be presumed that they are more difficult for users. In Study B, participants were aware of the no-error policy for progressing to the next level, which may have prompted them to allocate greater concentration to both the planning and execution of the task.

Given the limited duration of the training and the fact that no-one experienced the higher levels of difficulty in the training, it is possible that most of the participants did not reach their threshold level. However, although we still need to experiment with the training at the highest levels, we have gathered enough information to conclude that the progression of difficulty we have implemented is effective and would support adaptability throughout training, enabling older adults to tackle problems at the right level of difficulty.

These positive results are also corroborated by the results of the administered usability questionnaire. Indeed, the proportion of respondents who answered affirmatively to the question “Have you encountered any obstacle and/or difficulties during the exercise?” increased from 25% in study A to 45.4% in study B. Similarly, the proportion of respondents who answered affirmatively to the question “The difficulty level of the exercise increased gradually and progressively?” which was assessed with a 5-item Likert scale, increased from 3.4 (an almost neutral score) to 4.3 in Study B. In summary, the impression of users was that Version 2.0 of Weekend in Rome presented more challenging tasks with an increasing difficulty level.

In terms of usability and evaluation of ecological features, the results obtained in study B were similar to those obtained in study A. This can be seen as a positive result, which means that moving to a more complex planning system and more complex user interfaces did not have negative effects. However, it also means that further improvements are needed. For example, participants appreciated the introduction of short videos to present historical information about POIs, but found them repetitive. To address this, we added different videos at different levels of difficulty.

## 6. Discussion and Conclusions

The main contribution of this research is the validation of the fine-tuning of the planning task, which the presented results demonstrate to be effective. The Weekend in Rome prototype was significantly enhanced. We obtained these achievements by exploiting an advanced planner, ENHSP, which supports PDDL 2.1, a more expressive planning language. Thanks to fluent, it is possible to model more realistic features and to define exercises with higher difficulty. Moreover, minimization constraints for time and expenses can be enforced. New exercises can be created dynamically at a given difficulty level, allowing older adults to train their abilities in a variety of possible scenarios. In conclusion, ENHSP appears to be an effective tool in the implementation of planning training tasks.

Moreover, this research effort demonstrates the effectiveness of a participatory design approach in the development of cognitive training tasks for older adults. Two user studies were conducted to refine and tune an initial prototype, with study A involving and comparing two different age groups. The results presented in this paper show that these studies were essential for improving the features of a cognitive training to train problem-solving abilities. The use of a participatory design approach facilitates the rapid development of effective tasks. The integration of incremental iterations enabled the aggregation of user insights, culminating in the generation of a better product.

The primary outcome of pilot A highlighted the critical importance of progressively adjusting the difficulty stages of the exercises, which allowed us to refine this aspect. The results of pilot B demonstrate that, when following the adjustment of the difficulty levels of the exercises, there is a gradual increase, and they are deemed to be appropriate and sufficiently engaging for older adults. The incorporation of more intricate ecological features and problem-solving tasks did not adversely affect usability scores.

In comparison with previous research, we emphasize how automated planning facilitates the generation of numerous novel scenarios, as opposed to relying on a restricted set of predefined issues and solutions. This approach enables exercises to incorporate more characteristics typically observed in real-life planning activities, thereby enhancing the potential effectiveness of the proposed training for older adults.

Moreover, the use of automated planning addresses the theoretical issue of the practice effect by generating diverse and novel scenarios.

Future work will concern the improvement of the appearance of the training tasks, the addition of features to make them more realistic, the further improvement of the user interface, the introduction of unexpected events, and the addition of support for collaborative sessions. With consideration of the progression of difficulty levels, it was decided to reduce the number of consecutive correct attempts required to advance to the next level. This adjustment would allow more participants to reach the most challenging level, enabling them to train in cost minimization or path length reduction.

Finally, additional experiments are needed to assess the effectiveness of the proposed training program.

## Figures and Tables

**Table 1:** Means and standard deviations for the critical variables of the task. Standard deviations are presented in parentheses. The established threshold for significance was *p* < 0.05.

Variables	Young Group	Older Adults	*t*(20)	*p*
Sessions	2.09 (0.53)	4.27 (1.10)	5.89	<0.001
Not-achieved goals	8.73 (5.85)	13.82 (6.08)	2.00	0.059
Execution time	79.95 (31.94)	176.52 (66.66)	4.33	<0.001
Clicks on reservations	18.73 (11.81)	32.55 (14.67)	2.43	0.024
Clicks on goals	86.91(22.88)	110.72(43.98)	1.59	0.127

**Table 2:** Difficulty levels progression: Panel (a) presents a comparison of the minimal solution time in minutes (using a decimal notation) obtained by users to solve exercises at each difficulty level in the two studies. Since the success criteria changed in study B, we also report the minimal time obtained for passing exercises achieving all the goals, which is the criteria used in study B. The table also presents the number of users who were able to reach the difficulty levels. Panel (b) presents the average exercise generation time (in seconds) that the Trip Generator takes to create 30 exercises at the different difficulty levels, including the calls to the planner to verify exercise feasibility.

		Difficulty Levels								
(a)		1	2	3	4	5	6	7	8	9
**Study A**	80% goals	1.6	2.4	2.68	2.5	4.38	4.04	4.79	3.56	4.3
	100% goals	1.6	2.5	2.68	2.5	4.4	4.25	5.72	3.56	4.32
	N. users	11	11	11	11	11	11	11	11	10
**Study B**	100% goals	7.0	6.6	7.10	9.74	10.05	14.72	16.4	-	-
	N. users	11	11	11	11	10	6	3	-	-
(b)										
**Study B**	generation time	32	48	48	125	210	633	708	1492	2386

**Table 3:** Preliminary assessment: Means and standard deviations for BADS total score, subtests scores, and EPT scores administered at test, retest, and follow-up. The degrees of freedom (df) value in the ANOVA column is 2.18 for all rows. Standard deviations are presented in parentheses.

	T1: Test (N = 11)		T2: Re-Test (N = 11)		T3: Follow-Up (N = 10)		ANOVA	
Battery	M	(SD)	M	(SD)	M	(SD)	*F* Ratio	Time *p*
BADS	18.1	(2.4)	19.5	(2.0)	18.4	(2.7)	3.18	0.065
BADS: Rule Change	3.5	(0.7)	3.6	(0.5)	3.7	(0.7)	$2.96^{-30}$	1.000
BADS: Action Plan	3.8	(0.4)	4.0	(0.0)	3.9	(0.3)	1.00	0.387
BADS: Search Key	2.2	(1.3)	2.6	(1.0)	2.0	(1.1)	1.19	0.327
BADS: Time Estimates	3.0	(0.9)	3.3	(0.8)	2.8	(1.1)	2.11	0.150
BADS: Zoo Map	2.3	(0.9)	2.8	(0.9)	2.6	(1.1)	0.81	0.460
BADS: Six Elements Test	3.4	(0.9)	3.4	(0.8)	3.4	(0.7)	$1.24^{-29}$	1.000
EPT	11.7	(1.7)	10.6	(2.2)	11.6	(2.6)	1.89	0.180

## Data Availability

The original contributions presented in this study are included in the article. Further inquiries can be directed to the corresponding author.
